# Quantitative and Comparative Assessment of Recombinant Human β-Glucocerebrosidase Uptake Bioactivity Using a Stable hMMR-Expressing CHO Cell Model

**DOI:** 10.3390/molecules31020235

**Published:** 2026-01-10

**Authors:** Lyuyin Wang, Kaixin Xu, Ping Lyu, Xinyue Hu, Jing Li

**Affiliations:** 1 National Institutes for Food and Drug Control, State Key Laboratory of Drug Regulatory Science, NHC Key Laboratory of Research on Quality and Standardization of Biotech Products, NMPA Key Laboratory for Quality Research and Evaluation of Biological Products, NMPA Key Laboratory for Quality Research and Evaluation of Chemical Drugs, No. 31, Huatuo Road, DaXing District, Beijing 102629, China; wanglvyin@nifdc.org.cn (L.W.);; 2 School of Life Science and Technology, China Pharmaceutical University, No. 639, Longmian Road, JiangNing District, Nanjing 210009, China; 19850856619@163.com

**Keywords:** cellular uptake bioactivity, quality control, β-glucocerebrosidase, gaucher disease, method validation, receptor-mediated endocytosis

## Abstract

Inconsistent conclusions on the cellular uptake of recombinant human β-glucocerebrosidase (rhGCase) for Gaucher disease stem from a fundamental limitation of existing methods: their inability to generate complete and reliable dose–response curves. This critical flaw, stemming from susceptibility to various experimental variables, prevents accurate potency comparison across different rhGCase products. To address this, we developed a robust bioassay using CHO-K1 cells stably expressing the human macrophage mannose receptor (hMMR). Our method quantifies uptake by measuring the enzymatic activity of internalized rhGCase and consistently produces a classic sigmoidal dose–response curve. Comprehensive validation and mechanistic studies, including inhibition experiments with mannose, fucose, and mannose-6-phosphate, confirmed that uptake is specifically mediated by hMMR, with successful enzyme transport to endosomes/lysosomes. Applying this assay to three commercial products yielded results contrary to prior literature: imiglucerase demonstrated superior uptake activity to velaglucerase alfa. The proposed method represents a significant improvement over existing assays, providing a more accurate and reproducible means to evaluate cellular uptake bioactivity, which is crucial for the quality control of rhGCase therapeutics.

## 1. Introduction

Gaucher disease (GD), the most common lysosomal storage disorder, is caused by mutations in the GBA1 gene that lead to deficient activity of the lysosomal enzyme acid β-glucocerebrosidase (GCase) [1,2,3,4]. This results in the pathological accumulation of its substrate, glucosylceramide, within the lysosomes of macrophages. Enzyme replacement therapy (ERT) using recombinant human GCase (rhGCase) is the current standard of care, the efficacy of which fundamentally depends on the efficient internalization of the exogenous enzyme via the macrophage mannose receptor (MMR) and its subsequent targeted delivery to lysosomes [5,6,7,8,9,10,11,12].

The N-linked glycan structure of rhGCase is a critical determinant of this uptake efficiency [13]. Consequently, rhGCase products developed globally using different expression systems and glycoengineering strategies possess distinct glycan profiles: imiglucerase, produced in CHO cells, undergoes enzymatic trimming to expose core mannose residues; velaglucerase alfa, derived from human fibroblasts, is naturally rich in high-mannose glycans; and the newly developed velaglucerase beta, manufactured using a GnT1-knockout CHO cell line, achieves homogeneous mannose-terminal modification [14,15,16]. The coexistence and evolution of these products underscore the urgent need for a methodology capable of accurately quantifying and comparing their cellular uptake bioactivity—a critical quality attribute [17,18].

However, existing in vitro uptake assessment methods suffer from significant limitations, leading to contradictory conclusions across different studies. The approved ELISA method only detects the amount of internalized protein and cannot reflect the functional activity of the enzyme. Meanwhile, methods based on induced differentiated cell lines (e.g., PMA-induced U937 cells) are limited by unstable receptor expression, low detection sensitivity, and most critically, an inability to establish complete and reliable sigmoidal dose–response curves [17]. This flaw prevents the accurate calculation of key pharmacodynamic parameters such as EC_50_ and has directly resulted in fundamental discrepancies in the literature regarding the relative uptake efficiency of imiglucerase and velaglucerase alfa [19,20,21].

To resolve this long-standing ambiguity in the field and meet the stringent requirements of “Quality by Design” for biologics, this study developed and comprehensively validated a novel quantitative bioassay based on a stable CHO-K1 cell line expressing human MMR (CHO-hMMR). By integrating the detection of functional activity of the internalized enzyme with protein content normalization, this method successfully enables the generation of stable, reproducible, and complete dose–response curves. Using this validated platform, we directly compared the cellular uptake bioactivity of three major rhGCase products (imiglucerase, velaglucerase alfa, and velaglucerase beta). Notably, the quantitative results obtained in this study indicate that imiglucerase exhibits higher uptake activity than velaglucerase alfa, a finding contrary to the earlier report by Brumshtein et al. [17], thereby providing critical experimental evidence to clarify the contradictions in prior literature. The methodology established in this work offers a reliable and standardized tool for the development, quality control, and comparability assessment of rhGCase and related receptor-targeted enzyme therapies.

## 2. Results

### 2.1. Identification of CHO-hMMR Cells

**Figure 1:**
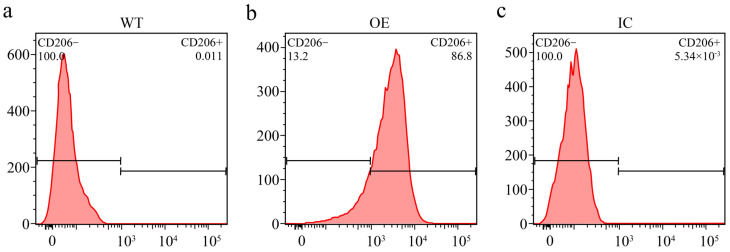
After overexpressing the mannose receptor on the surface of wild-type (WT) cells, flow cytometry was performed to quantify the receptor expression levels. (**a**) Detection of cell surface mannose receptor in WT CHO cells. (**b**) Detection of the cell surface mannose receptor in overexpressed (OE) CHO cells. (**c**) Isotype control (IC) detection of overexpressed cells.

To verify whether the stably transfected cells expressed hMMR, we used flow cytometry to identify and detect hMMR expression in the CHO-hMMR cell line. As shown in Figure 1b, flow cytometry showed that the positive rate of hMMR expression was 86.8% (Figure 1b), while the hMMR expression of wild-type cells was 0.011% (Figure 1a). The results showed that the monoclonal cell line successfully expressed the target gene hMMR, which can be used for subsequent optimization and validation.

### 2.2. Optimization of the Cellular Uptake Bioassay Based on CHO-hMMR Cells

**Figure 2:**
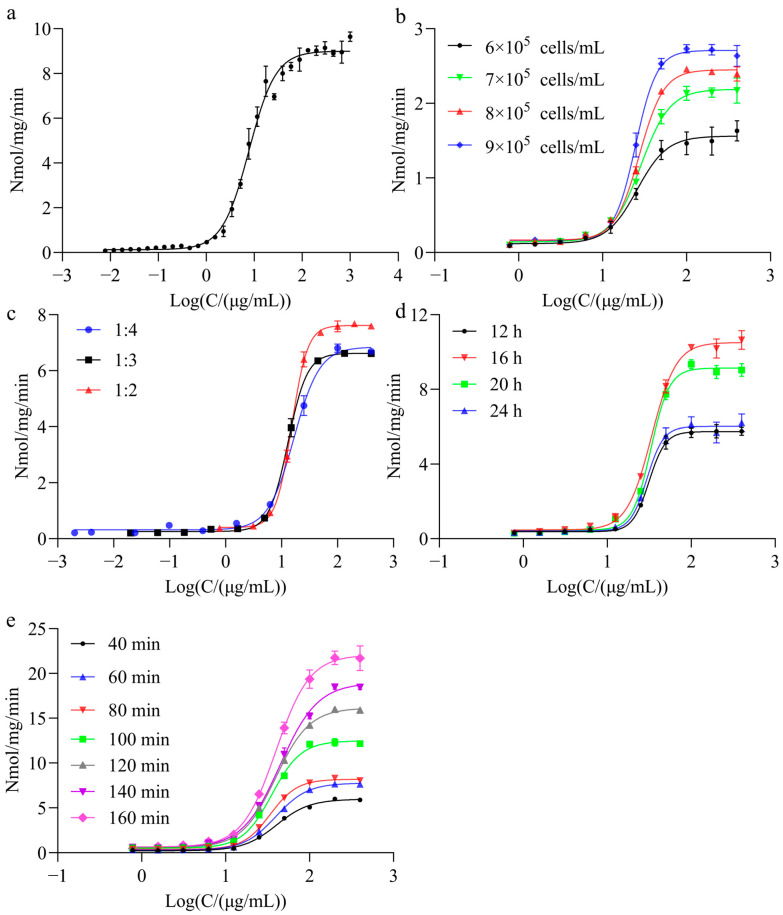
Optimization of recombinant human β-glucocerebrosidase (rhGCase) uptake activity assay based on CHO-hMMR cells. (**a**) Dose–response curve at an initial concentration of 900 μg/mL when cells were seeded at a density of 1.2 × 10^5^ cells per well. (**b**) Dose–response curve at an initial working concentration of 400 μg/mL, generated by plating cells at densities of 6 × 10^5^, 7 × 10^5^, 8 × 10^5^, and 9 × 10^5^ cells/mL to obtain 10 concentration points. (**c**) The shown dose–response curve was measured under 2-, 3-, and 4-fold serial dilutions of rhGCase. (**d**) The shown dose–response curve was measured at rhGCase incubation times of 12, 16, 20, and 24 h. (**e**) The shown dose–response curve was measured during the reaction of rhGCase with 4-methylumbelliferyl-β-D-glucopyranoside (4-MU-Glc) in cell lysate at 37 °C for 40, 60, 80, 100, 120, 140, and 160 min.

Four parameters, namely, dose-effect range of rhGCase, cell density, incubation time, and enzymatic reaction time, were examined individually to determine the optimal bioassay conditions. Upon stimulating CHO-hMMR cells with an rhGCase control at an initial concentration of 900 μg/mL, the dose–response curve showed a typical S-shape, as shown in Figure 2a. The optimal initial concentration was selected as the third concentration point on the upper plateau of the curve, with 400 μg/mL determined as the initial concentration for this method. Cell densities of 6 × 10^5^, 7 × 10^5^, 8 × 10^5^, and 9 × 10^5^ cells/mL were used for plating to detect the cellular uptake of the same rhGCase sample. As shown in Figure 2b, when the cell density was 8 × 10^5^ cells/mL, the four-parameter curve exhibited an R^2^ of 0.999, an S/N of 10.54, and a lower EC_50_ value of 12.83 μg/mL, confirming that 8 × 10^5^ cells/mL was the optimal cell plating density. The results of dilution factor optimization (Figure 2c) showed that only 2-fold serially diluted rhGCase incubated with cells yielded a typical S-shaped curve with favorable S/N and EC_50_ values. The drug stimulation time optimization (Figure 2d) indicated that stimulating cells with rhGCase for 16 h generated an S-shaped curve with a higher S/N (17.18) and a smaller EC_50_ value (40.34 μg/mL), indicating that 16 h was the optimal drug stimulation time. Optimization of the reaction time between the 4-MU-Glc substrate and cell lysate (Figure 2e) showed that a fluorescent substrate incubation time of 160 min resulted in a dose–response curve with uniformly distributed concentration points, clear upper and lower plateaus, and a higher S/N (26.48), indicating that the optimal substrate reaction time was 160 min.

### 2.3. Methodological Validation

#### 2.3.1. Specificity

**Figure 3:**
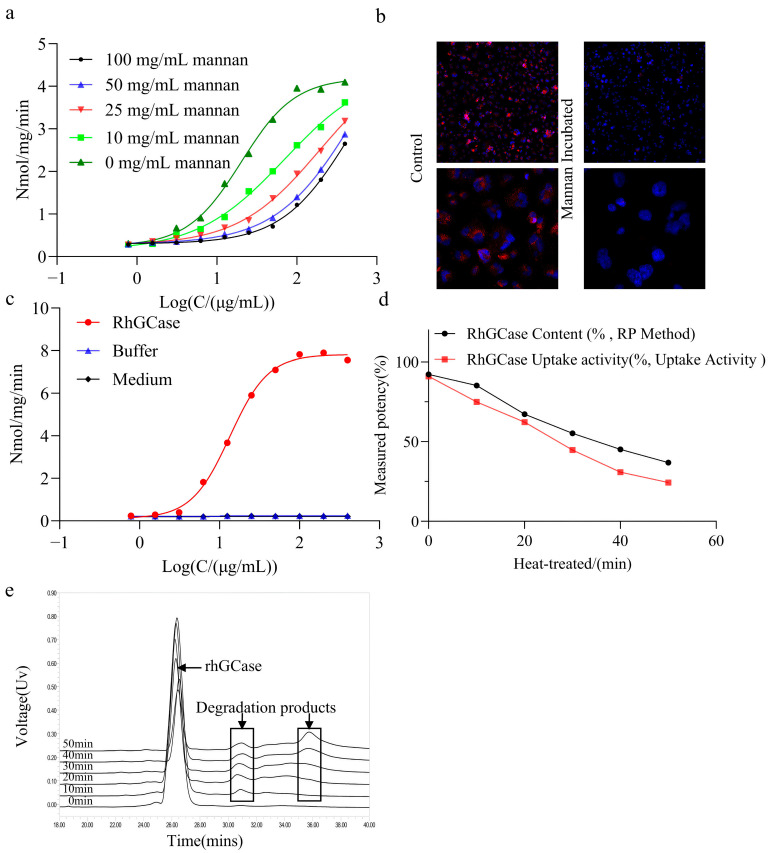
Specificity analysis of the rhGCase uptake activity assay. (**a**) Dose–response curves of rhGCase measured by the uptake activity assay in the presence of 100, 50, 25, and 10 mg/mL mannan. (**b**) Cells were incubated with rhGCase in the presence or absence of 40 mg/mL mannan (Man). Following incubation, cells were fixed with paraformaldehyde, incubated with a monoclonal anti-rhGCase antibody, treated with Alexa Fluor 488-conjugated secondary antibody (green, for rhGCase labeling), and stained with Hoechst 33342 (blue, for nuclear labeling). The parallel panels represent results from two independent experiments. Images were acquired at a magnification of 20×. Scale bars, 100 μm (not displayed in the figure). (**c**) Dose–response curves of formulation buffer, uptake medium, and rhGCase determined by the rhGCase uptake activity assay. (**d**) Relative potencies estimated by the rhGCase uptake activity assay and rhGCase sample contents measured by reverse-phase high-performance liquid chromatography (RP-HPLC) after incubation at 50 °C for different durations (min). (**e**) Reversed-phase chromatographic analysis of degradation products and rhGCase content in heat-damaged samples.

Specificity tests demonstrated uptake inhibition by 100, 50, 25, and 10 mg/mL mannan in a dose-dependent manner (Figure 3a). The inhibition was reversed by a gradual increase in uptake with lower mannan concentrations, demonstrating that CHO-K1-hMMR cells mediate rhGCase uptake through the mannose receptor. Concomitantly, cells were co-incubated with rhGCase, and high-content imaging was used to verify the receptor mediating rhGCase uptake. A strong uptake signal of the recombinant enzyme was detected in the control group without mannan, whereas the uptake signal of rhGCase was significantly attenuated in the experimental group with mannose added (Figure 3b). These results indicate that mannose can specifically inhibit rhGCase uptake, confirming that the cellular uptake of rhGCase is dependent on the mannose receptor. As shown in Figure 3c, neither the preparation buffer solution nor the measurement culture medium had an obvious dose-effect curve, whereas rhGCase showed a good four-parameter dose-effect curve, indicating that the method had good specificity. Additionally, compared with the untreated rhGCase reference standard, the relative uptake bioactivity of the treated reference standard decreased with increasing treatment duration, with percentages of 91.0%, 74.5%, 62.3%, 447%, 30.8%, and 24.4% after treatment at 50 °C for 10, 20, 30, 40, and 50 min (Figure 3d). The contents of rhGCase determined by reversed-phase chromatography were 92.3%, 85.2%, 67.2%, 55.2%, 45.17%, and 36.85%, respectively (Figure 3e). Therefore, the main component content of rhGCase gradually decreased with the increase in heating time, while the content of polar small-molecule substances gradually increased. This was consistent with the trend of rhGCase uptake activity obtained from the uptake activity assay, showing a positive correlation. The results showed that the established cellular uptake bioassay can sensitively detect the decrease in uptake activity caused by heating damage.

#### 2.3.2. Accuracy

The purpose of this study was to determine the closeness between the expected and observed values. The accuracy was measured at five levels of the analyte in the rhGCase reference standard, i.e., 0.50, 0.80, 1.00, 1.25, and 2.00 relative uptake activity units, by two analysts, and the results all passed the reliability test. Table 1 summarizes the results. The relative bias of the relative uptake measured within the 50–200% potency range of the bioassay was 0.3–5.4%, demonstrating that the relative potency measurements were close to the theoretical values, and the accuracy of the method was good.

#### 2.3.3. Intermediate Precision

Intermediate precision expresses within-laboratory variations, including different days, different analysts, and different cell passages. To assess the day-to-day, analyst-to-analyst, and cell passage-to-cell passage effects on precision, two analysts measured the relative uptake of the five aforementioned test solutions on 2 days. The intermediate precision of the method was evaluated using the geometric variation coefficient (GCV) at each potency level. The GCV (%) was calculated using the logarithmic values of the various potency level results, and the chi-square test was used to calculate the upper limit of the 95% confidence interval of GCV. The GCV ranged from 2.8% to 5.4%, and the corresponding 95% confidence interval upper limits were less than 6%, which proved the excellent precision of the bioassay (Table 2).

### 2.4. Linearity and Range

**Figure 4:**
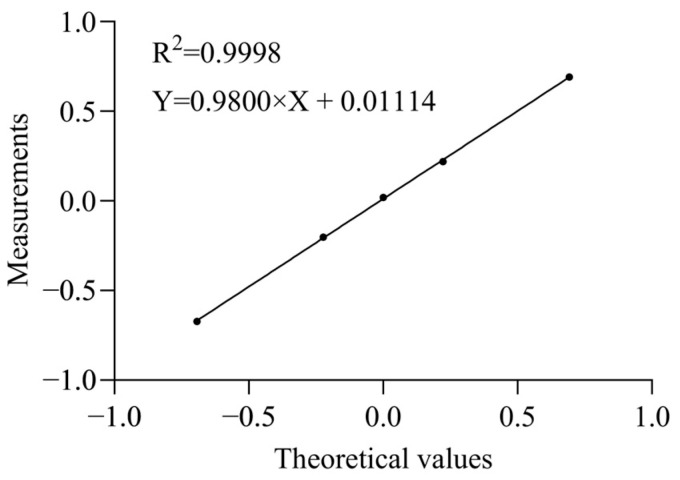
Linearity of the rhGCase uptake activity assay. In the rhGCase uptake activity assay, five samples at different relative uptake activity levels were analyzed, and a strong linear correlation was observed between the measured potency of rhGCase and the expected potency. Each data point represents the average of eight independent experiments.

The linearity of a method is the ability of the assay to obtain test results that are directly proportional to the sample concentration over a given range. The validation data plotted with the expected relative uptake on the X-axis and measured on the Y-axis revealed a regression slope of 0.9800 and a Y-intercept of 0.01114, with the correlation coefficient expressed with an R^2^ of 0.9963. Figure 4 shows the results of the regression analysis for the validation data. The slope was close to 1.0, and R^2^ was greater than 0.99, indicating that the linear fitting was good within the 50–200% potency range.

### 2.5. Reliability of the Assay Results

The Chinese Pharmacopoeia (2025 Edition), Volume IV, General Chapter 1431 “Statistical Methods for Biological Assays” specifies the requirements for each variation term in reliability tests: the regression term must be highly significant (*p* < 0.01), R^2^ must be ≥ 0.98, and the deviation from parallelism must be non-significant (*p* ≥ 0.05) [22].

Statistical analysis was performed for R^2^, the regression term, and the deviation from parallelism. The results showed that all R^2^ values exceeded 0.98 (minimum: 0.993), all regression terms were less than 0.01 (maximum: 1.345 × 10^−20^), and all deviations from parallelism terms were greater than 0.05 (minimum: 0.065). These findings meet the specifications of the Pharmacopoeia, demonstrating that the results obtained by this method are highly reliable.

### 2.6. Stability of Cell Passage

**Figure 5:**
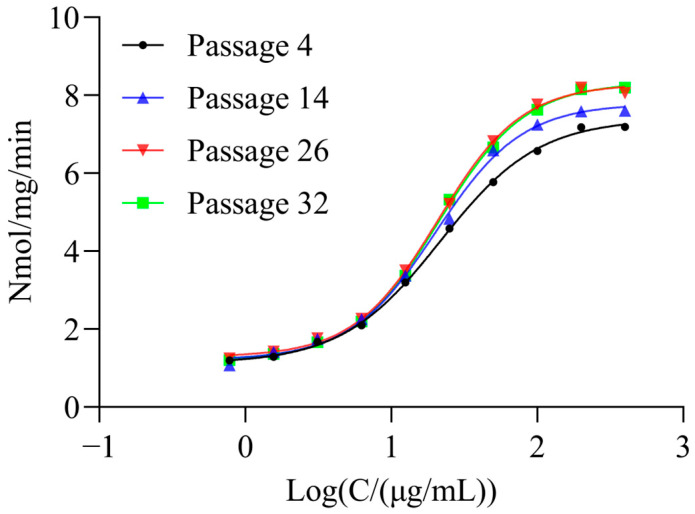
Passage stability analysis of CHO-K1-hMMR cells. Dose–response curves of the same rhGCase sample were determined using cells at passages 4, 14, 26, and 32.

The relative uptake activity of the same rhGCase test sample was determined using CHO-hMMR cells at four passages, with the results shown in Figure 5. The cells at all four passages could be fitted to typical S-shaped dose–response curves. The signal-to-noise (S/N) ratios of the curves were 6.82, 6.90, 6.61, and 6.86, respectively, and the EC_50_ values were 34.80, 39.02, 36.47, and 39.81 μg/mL, respectively. These results indicate that the CHO-hMMR cell line has good passage stability.

### 2.7. Application of the Cellular Uptake Bioassay

The relative uptake bioactivity of two batches of velaglucerase beta for injection (DP1, DP2) and two batches of velaglucerase beta bulk solution (DS1, DS2) was determined using the newly established and approved cellular uptake methods. Each batch was measured three times in parallel, and all results passed the reliability test. The relative standard deviation of the three repeated relative potency measurements of two batches of stock solutions and two batches of injections was less than 5% (Table 3), showing that the newly established method in this study had good repeatability in measuring the cellular uptake bioactivity of rhGCase and that it can be used as a routine quality control method for the cellular uptake of rhGCase products.

**Figure 6:**
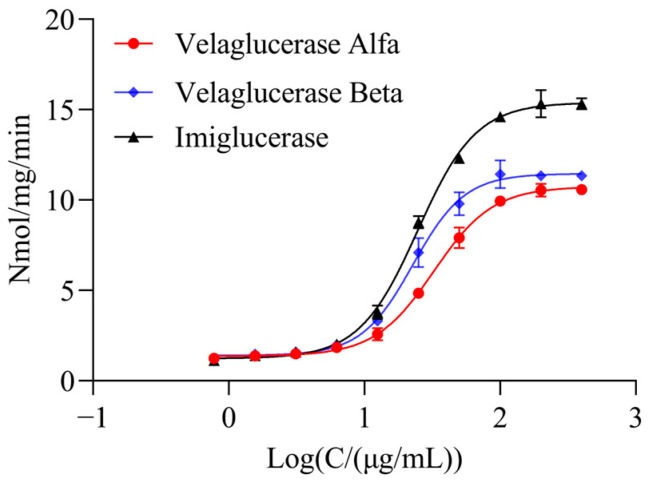
Drug uptake bioactivity was determined by the uptake activity assay. Dose–response curves of velaglucerase beta, imiglucerase, and velaglucerase alfa were measured using the proposed method.

The relative uptake bioactivities of imiglucerase, velaglucerase alfa, and velaglucerase beta for injection were determined using this method. As shown in Figure 6, all of the aforementioned rhGCase samples exhibited typical S-shaped dose–response curves. The EC_50_ values (from smallest to largest) were 60.7 μg/mL for imiglucerase, 70.2 μg/mL for velaglucerase beta, and 70.7 μg/mL for velaglucerase alfa. These results indicate that this method can effectively differentiate structural variations in rhGCase glycan modifications based on their dose–response characteristics.

### 2.8. Localization of rhGCase in CHO-hMMR Cells

**Figure 7:**
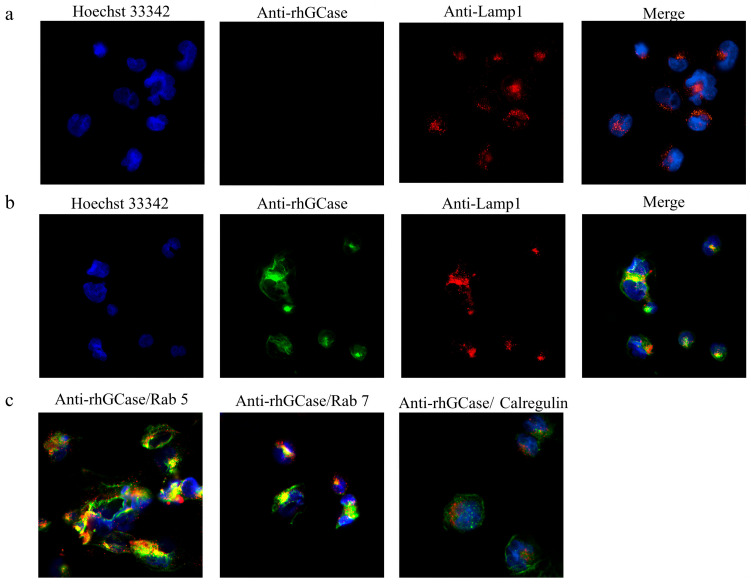
(**a**) Cells were fixed and incubated with a monoclonal anti-rhGCase antibody. Cells were treated with anti-rhGCase antibody (green) and stained with Hoechst 33342 (blue) for nuclear labeling. Images were acquired at a magnification of 20×. (**b**) After co-incubation with rhGCase, cells were stained with Hoechst 33342 (blue) and anti-rhGCase antibody (green) as in (**a**), along with anti-Lamp-1 antibody (red) for lysosome labeling. Merged images demonstrate rhGCase and Lamp-1 co-localization. Images were acquired at a magnification of 20×. (**c**) Merged images of cells co-incubated with rhGCase and stained for organelle markers Rab 5 (early endosome), Rab 7 (late endosome), and calreticulin (endoplasmic reticulum); anti-rhGCase antibody signals are shown in green, and organelle marker signals are shown in red. Images were acquired at a magnification of 20×. Scale bars, 100 μm (not displayed in the figure).

CHO-hMMR cells incubated with rhGCase were treated with rhGCase-specific monoclonal and lysosomal antibodies, followed by Hoechst 33342 staining. As shown in Figure 7a, no rhGCase signal was detected in untreated CHO-hMMR cells, whereas clear nuclear staining by Hoechst 33342 was observed. In rhGCase-treated cells (Figure 7b), distinct rhGCase signals (green) were co-localized with the lysosomal marker Lamp-1 (red), yielding a yellow hue from the overlap of green and red fluorescence. Similarly, green rhGCase signals showed co-localization with early (Rab 5) and late (Rab 7) endosomal markers (Figure 7c), but no co-localization of rhGCase signals with calreticulin was observed.

### 2.9. Receptors Involved in rhGCase Uptake by CHO-hMMR Cells

**Figure 8:**
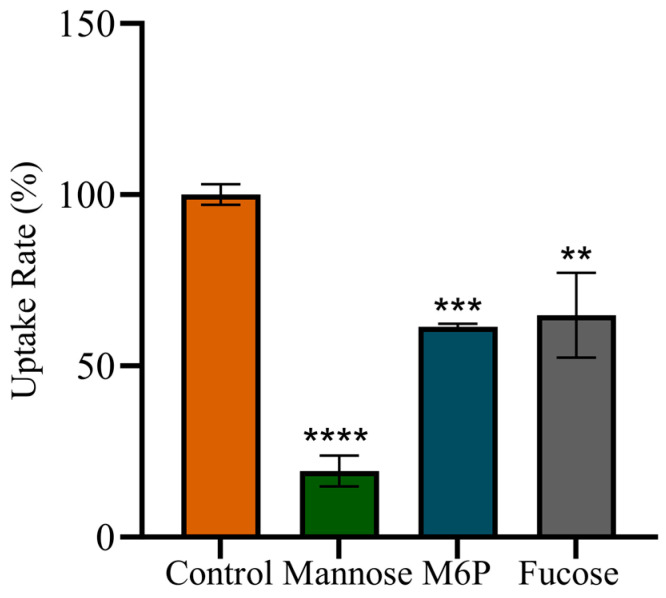
CHO-hMMR cells were co-incubated with D-mannose, D-mannose-6-phosphate (M6P), or fucose, and the uptake rate of rhGCase was determined. “Control” represents the group without sugar addition (uptake rate set as 100%). The results showed that D-mannose exhibited the lowest uptake rate (19%), while M6P and fucose showed relatively higher uptake rates (61% and 65%, respectively). Data are presented as the mean ± standard deviation from three independent experiments. Error bars are presented as the mean with standard deviation. Statistical analysis was performed with Student’s *t*-test. ** *p* < 0.01, *** *p* < 0.001, **** *p* < 0.0001.

To investigate the effects of fucose, D-mannose, and D-mannose-6-phosphate on the cellular uptake efficiency of rhGCase by CHO-hMMR cells, CHO-hMMR cells were co-incubated with the three aforementioned sugars. The results showed that all three sugars reduced the uptake rate of rhGCase compared to the control group (without sugar addition, uptake rate = 100%), but the degree of reduction differed significantly. Specifically, D-mannose led to the lowest rhGCase uptake rate (19%), indicating the strongest inhibitory effect; in contrast, D-mannose-6-phosphate (M6P) and fucose resulted in relatively higher uptake rates (61% and 65%, respectively), suggesting weaker inhibitory effects (Figure 8).

These results collectively support the conclusion that the cellular uptake of rhGCase by CHO-hMMR cells relies mainly on mannose receptors, with potential involvement of mannose-6-phosphate receptors to a lesser extent.

## 3. Discussion

Cellular uptake bioactivity is a critical quality attribute of rhGCase and its biosimilars, directly impacting the clinical efficacy of enzyme replacement therapy (ERT) [23]. However, due to the long-standing lack of a robust, quantifiable, and physiologically relevant in vitro evaluation method, conclusions regarding the relative uptake efficiency of mainstream products (e.g., imiglucerase vs. velaglucerase alfa) have often been contradictory across different studies. In this study, a novel bioassay based on CHO cells stably expressing human macrophage mannose receptor (hMMR, designated as CHO-hMMR cells) has been successfully established and validated, aiming to address this core methodological bottleneck in the field.

The core advantages of this method lie in its quantification, standardization, and physiological relevance. Compared with previous approaches using differentiation-induced cells (e.g., PMA-induced U937 cells) or primary monocytes from patients, the stable CHO-hMMR cell line ensures the uniformity of hMMR expression and inter-batch consistency of experiments [17,24], fundamentally eliminating data variability caused by fluctuations in receptor expression. More importantly, by integrating the detection of functional catalytic activity of internalized enzymes (based on 4-methylumbelliferyl-β-D-glucopyranoside hydrolysis) with total protein normalization, this method achieves, for the first time, the stable generation of a complete, classic sigmoidal dose–response curve for the rhGCase uptake process. This enables the accurate calculation of key pharmacodynamic parameters such as half-maximal effective concentration (EC_50_) and maximum effect (E_max_) [19], providing a reliable basis for direct, quantitative comparison of different products.

Application of this platform to three marketed rhGCase products yielded a clear conclusion: imiglucerase exhibits significantly higher cellular uptake activity than velaglucerase alfa. This result contradicts the earlier report by Brumshtein et al. We propose that this discrepancy likely stems from inherent limitations of the methodological approaches themselves [24]. Previous models suffered from insufficient stability of receptor expression and detection sensitivity, and failed to establish a complete dose–response relationship—factors that may have led to misjudgment of potency differences. Combined with existing glycoform analysis data (imiglucerase primarily presents short-chain Man3-type glycans with a higher degree of fucose modification, while velaglucerase alfa is dominated by long-chain Man6-Man9-type high-mannose glycans) [7,25], our results highlight the critical role of glycan structure in regulating receptor binding and internalization efficiency. Shorter Man3 glycan chains may reduce steric hindrance, facilitating multivalent binding to cell surface hMMR and thereby explaining imiglucerase’s higher uptake efficiency [26,27,28]. This lays a solid foundation for the future application of this method in systematic investigations of the “glycoform structure-uptake activity” structure-activity relationship, with the potential to guide rational glycosylation design of next-generation products.

Further mechanistic studies, particularly sugar inhibition experiments, provided a clear definition of the uptake pathway. Results demonstrated that rhGCase uptake by CHO-hMMR cells is primarily mediated by hMMR (D-mannose exhibited the strongest inhibitory potency) [29], with the mannose-6-phosphate (M6P) receptor pathway playing a minor role and fucose showing a relatively weaker inhibitory effect [30]. Notably, the literature’s conclusions regarding the affinity of fucose for hMMR are inconsistent. In this study, fucose exhibited weaker inhibitory potency than mannose in the whole-cell model—a finding that differs from some in vitro binding experiments based on purified receptor domains (e.g., CRD4) [31]. This discrepancy underscores the unique value of evaluating receptor-ligand interactions in the context of intact cell membranes, as whole-cell models better reflect physiological complexities such as collaboration between multiple receptor domains, membrane fluidity, and endocytic regulatory networks. Thus, our method serves not only as a quality control tool but also as a superior platform for more authentically assessing the biological outcomes of glycoform-receptor interactions.

The primary significance of the established method is that it provides a standardized, verifiable, and robust tool for quality control, process optimization, and comparability studies of rhGCase products. It can sensitively detect changes in product activity caused by process modifications or storage conditions, aligning with the advanced concept of “Quality by Design.” Secondly, the value of this platform extends beyond quality control for a single product: it offers an ideal in vitro model for exploring how glycosylation modifications precisely regulate the intracellular delivery efficiency of hMMR-targeted biological macromolecules, with broad applicability to early-stage research and development of other ERT drugs or receptor-targeted therapeutics [25].

Naturally, this study has inherent limitations. The most prominent limitation lies in the selected cell model itself. Although CHO-hMMR cells stably express key receptors and fully recapitulate the endocytosis-lysosome targeting process, they are not the physiological target cells of rhGCase—tissue-resident macrophages. Differences may exist between the two cell types in terms of receptor repertoire, regulation of endocytic pathways, and intracellular microenvironment. Therefore, this method is primarily positioned as an excellent in vitro quality control and screening tool. While the relative activity trends it measures hold high reference value, validation in more complex preclinical models (e.g., animal models or primary macrophage models) is still required for the final prediction of in vivo pharmacodynamics and clinical outcomes. Future research directions include verifying the predictive relevance of this assay in more physiologically relevant cell models and using this platform for high-throughput screening of the effects of different glycoengineering strategies on the delivery efficiency of various receptor-targeted protein drugs, thereby constructing a more comprehensive “structure-function” relationship map.

In conclusion, an innovative in vitro bioassay has been developed, which successfully addresses long-standing quantitative evaluation challenges in the rhGCase field and opens new avenues for structure-based optimization of drug potency. By revealing new insights into the inhibitory potency of fucose, this study also emphasizes the importance of conducting mechanistic research in physiologically relevant systems.

## 4. Materials and Methods

### 4.1. Cell Lines and Reagents

CHO cells expressing hMMR were supplied by CANbridge Pharmaceuticals Inc. (Shanghai, China) and were cultured in CD CHO medium supplemented with 4 mM L-glutamine, 200 μg/mL zeocin, and 1% hypoxanthine sodium-thymidine additive. The BCA protein assay reagent and M-PER™ Mammalian Protein Extraction Reagent were purchased from ThermoFisher Scientific (Shanghai, China). The 4-MU-Glc and 4-MU were purchased from Sigma-Aldrich (Beijing, China). Anti-LAMP1, anti-RAB5, anti-RAB7, and anti-calreticulin rabbit polyclonal antibodies were purchased from ThermoFisher Scientific (Shanghai, China). Alexa Fluor 488 goat anti-rat IgG Ab, Alexa Fluor 594 goat anti-rabbit IgG Ab, and Hoechst 33342 were obtained from Thermo Fisher Scientific (Shanghai, China). Anti-rhGCase monoclonal rat antibody and anti-CD206 (mannose receptor, MR) monoclonal rabbit antibody were purchased from Abcam (Shanghai, China) and Thermo Fisher Scientific (Shanghai, China), respectively. Velaglucerase beta for injection and the stock solution were obtained from CANbridge Pharmaceuticals Inc. (Shanghai, China). Imiglucerase for injection was obtained from Sanofi Pharmaceutical Co., Ltd. (Ireland, UK). Velaglucerase alfa for injection was obtained from Vetter Pharma-Fertigung GmbH & Co. KG (Ravensburg, Germany). Fucose, D-mannose, and D-mannose-6-phosphate from Sigma-Aldrich (Beijing, China).

### 4.2. Flow Cytometry Analysis

Flow cytometric analysis was performed to investigate hMMR expression in CHO-MMR cells. The CHO-MMR cells were collected by centrifugation at 1000 rpm for 5 min, before washing and resuspending to 1 × 10^6^ cells/mL with ice-cold 2% fetal bovine serum (FBS)/PBS. Cell suspensions were stained with APC-labeled MMR antibody for 30 min at 4 °C in the dark. hMMR fluorescence was determined using the FL3 channel on a fluorescence-activated cell sorter (BD Biosciences, San Jose, CA, USA). The percentage of hMMR expression was calculated by using cells without fluorescent labeling as the control.

### 4.3. Cellular Uptake Bioassay

CHO-hMMR cells were seeded in 96-well microtiter plates (Corning, NY, USA) at 80,000 cells per well in 150 μL assay medium (RPMI 1640 medium with 5% FBS and 1 mM DTT) per well and then incubated at 37 °C with 5% CO_2_ for 1 h. The rhGCase sample was serially diluted in 2-fold increments to generate 10 concentration points, with an initial working concentration of 400 μg/mL. Then, 50 μL of the dilutions was added to each well in the plate, which was incubated at 37 °C with 5% CO_2_ for 16 h. Next, we added 50 μL of 0.25% trypsin into each well in the plate to disperse the cells. Then, 200 μL of digestion stop solution (DPBS with 4% FBS) was added to each well, before transferring the cells to 96-well conical-bottom microtiter plates and briefly centrifuging. After centrifugation, the cells were washed with DPBS and then lysed (lysis buffer: MPER mammalian protein extractant with 1% Halt protease inhibitor mixture) at 37 °C for 1 h. The internalized rhGCase was quantified using the 4-MU-Glc enzymatic activity assay. Briefly, 4-MU was diluted to 8, 2.86, 1.02, 0.36, 0.13, 0.05, and 0.02 nM with sodium acetate buffer (pH = 4.8) containing Triton X-100 to prepare a 4-MU standard curve solution, which was added to a black 96-well plate at 50 μL/well. The cell lysate was transferred to a black 96-well plate at 25 μL/well, before adding 5 mM 4-MU-Glc substrate solution at 25 μL/well. The black 96-well plate was incubated at 37 °C with 5% CO_2_ in the dark for 160 min, after which 125 μL of stop solution (1 M glycine solution, pH = 10.5) was added to each well, and the relative fluorescence value was read according to the preset parameters (excitation wavelength: 360 nm, emission wavelength: 460 nm, bottom reading) in a SpectraMax M5/M5e microplate reader (Molecular Devices, LLC, San Jose, CA, USA). The specific activity (nmol/mg/min) of the rhGCase standard and test samples at each concentration was calculated using the measured 4-MU concentration (nmol/mL) obtained from enzymatic reactions and the protein concentration (mg/mL) obtained via the BCA method (performed according to the manufacturer’s protocol). A dose–response curve was generated by plotting the specific enzyme activity (nmol/mg/min) of both the rhGCase standard and test samples at each concentration point against their corresponding concentrations (ng/mL), followed by four-parameter logistic curve fitting. The relative activity of the samples was calculated as the percentage of the EC_50_ value of the standard relative to that of the samples using the constrained fitting model. The results were analyzed using SoftMax Pro software 7.1.2.

### 4.4. Specificity

The assay specificity was demonstrated through antagonism of the mannose receptor using free mannan in the media. To demonstrate specificity, soluble mannan at different concentrations (100, 50, 25, and 10 mg/mL) was utilized in the assay to occupy the mannose receptor in CHO-hMMR cells to antagonize the rhGCase uptake. Meanwhile, high-content imaging technology was used to analyze the receptors involved in rhGCase uptake: CHO-hMMR cells were seeded in 96-well plates and divided into two treatment groups upon reaching an appropriate density. The control group was incubated with rhGCase alone, whereas the experimental group was simultaneously co-incubated with rhGCase and 40 mg/mL mannan. After incubation, both groups of cells were processed using identical procedures: fixed with 4% paraformaldehyde, permeabilized with 0.1% Triton X-100, blocked with FBS, and then incubated overnight with GBA primary antibody. The following day, the cells were incubated with Alexa Fluor 488-conjugated secondary antibody at room temperature, and a high-content imaging system was used to acquire fluorescent images. Each group was set with three replicate wells, and intergroup differences were analyzed using statistical methods. Because excipients and degradants are typically investigated to assess specificity, the rhGCase preparation buffer solution, the measurement culture medium, and heat-degraded rhGCase (heated at 50 °C for 50 min) were used to treat the CHO-hMMR cells. All samples were measured using the cellular uptake assay described above. In addition, reversed-phase high-performance liquid chromatography was used to determine the rhGCase content in the samples heated at 50 °C.

### 4.5. Accuracy, Precision, and Linearity

The rhGCase reference standard was pre-diluted to 400 μg/mL with assay medium as the control for validation. Serial solutions with initial concentrations of 200, 320, 400, 500, and 800 μg/mL were prepared with assay medium, serving as test solutions for five potency levels (50%, 80%, 100%, 125%, and 200%). Two analysts used two cell passages to determine the relative potencies of the five test solutions within 2 days, with eight replicates per potency level, and calculated the geometric means. The relative accuracy was evaluated using relative bias (RB = measured relative potency/theoretical relative potency × 100%). To assess the intermediate precision of the method, the geometric coefficient of variation (GCV, %) and the upper limit of the 95% confidence interval were calculated based on the logarithmic values of the determination results at each potency level. Linear regression was performed by plotting the natural logarithm of the theoretical values (x) against that of the measured values (y) for the five potency levels using the least-squares method to examine the linearity and range of the method [22].

### 4.6. Assay Reliability

The test data were analyzed using SoftMax Pro software, and graphs were plotted using GraphPad Prism software 9.5. The logarithm (base 10) of the rhGCase concentration was set as the abscissa, and the specific enzyme activity (nmol/mg/min) was set as the ordinate to plot a four-parameter dose–response curve described by the equation: y = (A − D)/[1 + (x/C)^B^] + D, where A is the upper asymptote, D is the lower asymptote, C is the half maximal effective concentration (EC_50_), and the signal-to-noise ratio (S/N) = A/D. Referring to the “Four-Parameter Regression Calculation Method” in General Chapter 1431 of Volume IV of the Chinese Pharmacopoeia (2025 edition), the results were subjected to reliability testing using analysis of variance (ANOVA) and F-test. The acceptance criteria were as follows: the fitted four-parameter curve had an R^2^ ≥ 0.98, the regression term was highly significant (*p* < 0.01), and the deviation from parallelism was not significant (*p* ≥ 0.05). Only results that passed the reliability test were used to calculate the relative uptake activity (%) as (control EC_50_/sample EC_50_) × 100% [22].

### 4.7. Stability Test of Cell Passage

Cells at passages 5, 13, 24, and 34 were selected as the experimental subjects. The same batch of rhGCase was added to determine the uptake activity and to investigate the stability of cell passages.

### 4.8. Application of the Cell Uptake Bioassay

The uptake activity of two batches of velaglucerase beta for injection, two batches of velaglucerase beta bulk solution, one batch of imiglucerase for injection, and one batch of velaglucerase alfa for injection were determined using the proposed method.

### 4.9. Localization of rhGCase in CHO-hMMR Cells

CHO-K1-hMMR cells were seeded at 10,000 cells/well in black 96-well plates coated with poly-L-lysine (Phenoplate, Corning, NY, USA). After adding rhGCase, the plates were incubated at 37 °C in a 5% CO_2_ incubator for 8 h. The culture medium was aspirated, and the cells were fixed with 4% paraformaldehyde at room temperature in the dark for 30 min, followed by four washes with DPBS. The cells were permeabilized with 0.1% Triton X-100 in DPBS for 30 min and washed three times. Subsequently, 2% bovine serum albumin in DPBS was added for 30 min at room temperature, followed by incubation with FBS for 60 min to block non-specific binding. After aspirating the blocking solution, the primary antibodies against lysosomes, endoplasmic reticulum, early endosomes, and late endosomes, and rhGCase primary antibodies were added to different wells and co-incubated at 4 °C on a shaker for 14 h. After four washes with DPBS, the cells were incubated for 1 h at room temperature in the dark with 0.02% goat anti-rabbit cross-adsorbed secondary antibody in FBS solution and 0.02% goat anti-mouse cross-adsorbed secondary antibody in FBS solution. Following immunostaining, the cells were washed three times with DPBS, stained with Hoechst 33342 solution, and subjected to high-content imaging. Images were analyzed using an Operetta CLS high-content cell imaging analysis system, which is equipped with four lasers and a transmission light source, including 405 nm violet diode excitation, 488 nm multi-line argon blue excitation, 543 nm helium-neon green excitation, and 633 nm helium-neon red excitation [32,33]. To minimize fluorescence crosstalk, sequential scanning was performed using an FV500 confocal microscope. For focusing, the blue nuclear staining by Hoechst 33342 was used to determine the focal plane. For immunofluorescence staining, three replicate samples were set for each condition, and three images were acquired per sample.

### 4.10. Inhibition Experiment

Fucose, D-mannose, and D-mannose-6-phosphate were individually dissolved in assay medium to prepare 50 mM stock solutions. The rhGCase reference standard was diluted to a concentration of 400 μg/mL using the aforementioned assay medium.

CHO-hMMR cells were seeded in 96-well microtiter plates at a density of 80,000 cells per well in 150 μL of assay medium per well. The plates were incubated at 37 °C with 5% CO_2_ for 1 h to allow cell adhesion. Subsequently, the cells were divided into three experimental groups (fucose group, D-mannose-6-phosphate group, mannose group) and one positive control group (no-inhibitor group). For all groups, 50 μL of 400 μg/mL rhGCase was first added to each well; thereafter, 50 μL of the corresponding 50 mM sugar stock solution was added to each well in the experimental groups, while 50 μL of assay medium was added to each well in the control group. Each group was set with triplicate wells, and the plates were incubated at 37 °C with 5% CO_2_ for 16 h.

Subsequent cell processing was performed strictly in accordance with Section 2.3. To evaluate the effect of the three sugars on rhGCase cellular uptake, the uptake rate was calculated using the formula: specific activity of rhGCase in inhibitor-treated groups/specific activity of rhGCase in the no-inhibitor control group.

## 5. Conclusions

This study established a novel assay for the quantitative assessment of the cellular uptake bioactivity of recombinant human β-glucocerebrosidase (rhGCase), based on CHO cells stably expressing the human macrophage mannose receptor (hMMR). The method reliably generates complete dose–response curves and confirms that the uptake process is primarily mediated by hMMR, with the internalized enzyme correctly trafficked to endosomes and lysosomes, thereby ensuring its catalytic function.

Applying this assay to compare three rhGCase therapeutics revealed that imiglucerase exhibits higher uptake activity than velaglucerase alfa. Inhibition experiments further indicated that the process is dominated by the mannose receptor pathway, with the mannose-6-phosphate receptor pathway playing a secondary role.

The assay developed in this study not only provides a reliable basis for clarifying differences in uptake activity among similar products but also offers a mechanism-relevant analytical tool for the quality evaluation and comparability assessment of rhGCase and related enzyme replacement therapies.

## Figures and Tables

**Table 1:** Validation of the accuracy of the uptake activity assay for detecting the relative potency of rhGCase (recombinant glucocerebrosidase).

Potency Level	Run	Relative Potency			Relative Bias		
		Mean	Upper Confidence	Lower Confidence	Mean	Upper Confidence	Lower Confidence
50%	8	51.10%	52.76%	49.45%	2.2%	5.4%	1.1%
80%	8	81.80%	83.32%	80.28%	2.3%	4.0%	0.3%
100%	8	102.01%	105.59%	98.43%	2.0%	5.5%	1.6%
125%	8	124.74%	127.14%	122.33%	0.2%	1.6%	2.2%
200%	8	199.79%	204.48%	195.10%	0.1%	2.2%	2.5%

**Table 2:** Intermediate precision validation of the uptake activity assay.

Potency Level	Run	GSD	CIGSD	GCV	CIGCV
50%	8	1.0494	1.0905	4.9%	9.0%
80%	8	1.0279	1.0507	2.8%	5.1%
100%	8	1.0536	1.0983	5.4%	9.8%
125%	8	1.0291	1.0528	2.9%	5.3%
200%	8	1.0357	1.0651	3.6%	6.5%

GSD: geometric standard deviation; CIGSD: 95% upper confidence limit of GSD; GCV: geometric variation coefficient; CIGCV: 95% upper confidence limit of GCV.

**Table 3:** Uptake activities of the four batches of rhGCase drug stock solutions and injections, as determined by the uptake activity assay.

Samples	Mean	RSD
DS1	104.83%	2.36%
DS2	102.00%	4.10%
DP1	101.40%	0.74%
DP2	104.60%	3.67%

## Data Availability

Data will be made available on request.

## Abbreviations

The following abbreviations are used in this manuscript:

GD	Gaucher disease
LSD	Lysosomal storage disorder
ERT	Enzyme Replacement Therapy
GnT1	Gene encoding N-acetylglucosaminyltransferase 1
rhGCase	Recombinant human β-glucocerebrosidase
ELISA	Enzyme-linked immunosorbent assay
PMA	Phorbol myristate acetate
EC_50_	Half-maximal effective concentration
E_max_	Maximum effect
hMMR	Human macrophage mannose receptor
4-MU-Glc	4-methylumbelliferyl-β-D-glucopyranoside
4-MU	4-methylumbelliferone
BCA	Bicinchoninic acid
WT	Wild-type
OE	Overexpressed
IC	Isotype control
RP-HPLC	Reverse-phase high-performance liquid chromatography
GCV	Geometric variation coefficient
GSD	Geometric standard deviation
CIGSD	95% upper confidence limit of GSD
CIGCV	95% upper confidence limit of GCV
M6P	D-mannose-6-phosphate
PBS	Phosphate-buffered serum
FBS	Fetal bovine serum
S-shaped	Typical sigmoidal
IC_50_	Half-maximal inhibitory concentration
SPR	Surface plasmon resonance
ER	Endoplasmic reticulum
